# Physicians’ perspectives on family caregivers’ roles in elderly cancer patients’ therapies: a qualitative, interview-based study

**DOI:** 10.1007/s00520-023-07857-6

**Published:** 2023-06-09

**Authors:** Andreas Heidenreich, Susanne Elsner, Frank Wörler, Joachim Hübner, Christina Schües, Christoph Rehmann-Sutter, Alexander Katalinic, Frank Gieseler

**Affiliations:** 1 Institute of Social Medicine and Epidemiology, University of Luebeck, Luebeck, Germany; 2 Institute for History of Medicine and Science Studies, University of Luebeck, Luebeck, Germany; 3 Clinic for Hematology and Oncology, University Hospital Schleswig‐Holstein (UKSH), Luebeck, Germany

**Keywords:** Family caregivers, Clinical communication, Therapy decision-making

## Abstract

**Purpose:**

Clinical communication and facilitating informed and sound medical decisions become challenging as patients age and suffer from age-associated impairments. Family caregivers are perceived as essential actors in addressing these challenges. Here, we explore physicians’ perspectives on family caregivers’ roles and their involvement in consultations and therapy decision-making situations of elderly cancer patients.

**Methods:**

We examined 38 semi-structured interviews with physicians from different specialities (oncologists, non-oncology specialists, and general practitioners) in Germany who treated elderly cancer patients. Data were analyzed using reflexive thematic analysis.

**Results:**

We identified five general and distinct perspectives on the involvement of family caregivers in the therapy process. Family caregivers are seen as (1) translators of medical information; (2) providers of support for the patient; (3) providers of information about the patient; (4) stakeholders with relevant points of view regarding the treatment decision; or (5) individuals who have a disruptive influence on the consultation. The interviewed physicians rarely involved family caregivers closely in consultations.

**Conclusions:**

Although physicians frequently attribute supportive roles to family caregivers, they rarely include them in consultations. Previous studies have found that a triadic setting is often better suited to agreeing upon a patient-centered and needs-based treatment decision for older cancer patients. We infer that physicians too rarely recognize the potential importance of family caregivers. Educators should further integrate family caregiver involvement and its implications in general medical education and professional training.

## Introduction

As patients age and suffer from age-associated impairments, therapy decision-making and communication in a clinical setting become increasingly challenging for both patients and physicians [1–8]. It often remains unclear whether the doctor and patient understand one another as each intended. A doctor-patient dyad is not always the proper setting in which an informed patient’s will can be successfully communicated [9]. The treatment process in cancer is particularly complex and involves a great deal of organizational effort. In Germany, the lack of healthcare professionals coordinating the complex treatment process was found to be a cause for concern [10]. The involvement of family members, confidants, and caregivers—hereafter referred to as family caregivers (FCGs)—in supporting the patient during therapy and follow-up is very important for successful treatment and patient satisfaction [11–13]. Therefore, it is reasonable to include FCGs in the therapy process to plan and organize treatment and reach a therapy decision that meets the needs and wishes of the patient. It is especially beneficial to involve FCGs in situations in which clincians recommend treatments that are more aggressive than patients were initially wishing for or expecting [14, 15].

A previous study based on interviews with elderly cancer patients and FCGs in Germany examined the latters’ influence on the therapy decision-making process [9]. The authors found that FCGs influence the therapy decision to varying degrees. These findings are consistent with similar recent studies in other locations [16, 17]. Depending on the family situation and other factors, FCG involvement in therapy decision-making might either be to the benefit or detriment of the patient’s autonomy. Overwhelmed patients may require advocacy to address their wants and needs adequately in consultations. On the other hand, strong FCG involvement can lead to physician and FCG discussions *about* the patient, excluding them from active participation during the consultation and potentially violating the patient’s autonomy over therapy decisions. In any case, the physician should be aware that other parties can potentially influence the patient’s decision. Thus, if the physician wants to fully understand the patient’s decision, it is necessary to be mindful of possible external influences. However, it may also be the patient’s desire to put the needs of close people at the center of their thinking and actions. This interrelation of patients’ and FCGs’ wishes is not necessarily a threat to the patient’s free will [18]. The study mentioned above also indicated a discrepancy between patients’ wishes regarding FCG involvement and their actual participation in relevant discussions [9]. This is at odds with the recommendations of major German oncology guidelines to accommodate the patient’s wish to include FCGs in the therapy process [19–23]. In addition, there are qualitative interview studies that address the views of physicians and other health professionals on the involvement of FCGs [24, 25]. However, Laidsaar-Powell and colleagues have rightly recognized that much of the relevant literature on FCG involvement in cancer therapy decision-making is very dated. Thus, “results may not reflect the current state of consultation communication and decision-making” [16] or do not focus on cancer therapy decision-making. We concur with their judgment that further research using qualitative methods is needed. We use semi-structured interviews with clinicians involved in treating elderly cancer patients to examine their views regarding the involvement of FCGs in the cancer treatment process and to what extent they include FCGs in consultations and decision-making situations. The findings shall inform future debates on the potential benefits and drawbacks of FCG involvement regarding the quality of care for older cancer patients.

## Methods

### Study design

To capture the heterogeneity of care for older cancer patients, we sought oncologists, non-oncology specialists, and general practitioners to achieve a balanced representation of each specialist group. Both inpatient and outpatient clinicians were considered. Physicians were recruited through convenience sampling with the Medical Association of Schleswig–Holstein’s support, which provided eligible persons’ contact details. Participants were queried in semi-structured, guideline-based interviews on FCG involvement in cancer therapy of elderly patients, and received financial compensation of EUR 40.00.

Specific interview guides were prepared for the respective groups. The broad and balanced sample aimed to cover a wide range of relevant contexts. Participants’ responses were qualitatively analyzed to identify and characterize overarching themes of FCG inclusion in cancer therapy using a reflexive thematic analysis (RTA) approach [26].

This study was part of a larger project on cancer therapy and guideline adherence in elderly patients (70 years and older). Findings from interviews with patients and FCGs are published elsewhere [9, 27, 28]. The ethics committee of the University of Luebeck approved the overarching project before the start of the study (decision: 5 October 2017, file: 17–288).

### Interview guides

We developed different interview guides for oncologists, non-oncology specialists, and general practitioners to reflect each discipline’s roles in the treatment process. The interview guides were designed in a highly reflective and discursive process, involving a multidisciplinary team of physicians, social scientists, ethicists, and psycho-oncologists. Research experience and clinical expertise were equally incorporated into the guides. The interview guides were designed for semi-structured interviews and related to thematic foci but intended to allow interviewed clinicians to set their focal points. The main topics of the interview guide are presented in box 1.

**Box 1:** Overview of key questions in interview guidelines

• Challenges in the care of elderly oncology patients • Attitudes and approaches to the clinical situation • The complexity of care of elderly cancer patients • Special considerations in the treatment of cancer in elderly patients
• Quality of life vs. life prolongation and “medical futility” • Dealing with differences of opinion in doctor-patient discussions • Role of FCGs in the therapy process and therapy decision-making

Each of the developed guides was tested on representatives of the respective target group for comprehensibility and feasibility.

### Data collection and data processing

The interviews were conducted either by telephone or at the physician’s premises. All interviewees consented to the interview being recorded and transcribed verbatim and agreed for their interviews to be used for research purposes. All mentions of people and places were obscured during the transcription process.

### Data analysis

We analyzed the data using the RTA approach [26, 29, 30]. This analytical framework is deemed particularly useful to understand participants’ “views, perspectives, and experiences.” [29] As the project was primarily exploratory in character and was not focused on a specific research question from the outset, we refrained from linking data collection and data analysis in the sense of an iterative, theoretical sampling process [31, 32]. RTA has been found well-suited for analyzing pre-existing data sets for themes and patterns that might not have been anticipated in the original data collection process [29].

Initially, we screened the interviews for mentions and assessments of the characteristics of older patients’ treatment and their specific needs in order to familiarize ourselves with the data (A.H., S.E., F.G.). We subsequently focused on coding interviewees’ expressions and remarks on the significance and the roles of FCGs in cancer therapy, consultation settings, and therapy decision-making processes (A.H., S.E., F.G.). The generated themes were evaluated, critiqued, and validated reflexively (A.H., S.E., F.W., J.H., F.G.). The validated themes were then used to develop a condensed but accurately descriptive typology that maps the spectrum of physicians’ perspectives on the inclusion of FCGs (A.H., S.E., F.G.). Finally, we compiled indicative quotations to illustrate the types identified, as presented below (A.H., F.G.). All interviews were conducted in German. Quotes from these interviews shown below have been translated into English under guidance of a bilingual native speaker to ensure that the meaning of the statements is represented accurately.

## Results

Between February 2018 and July 2018, we recorded 38 semi-structured interviews with physicians from various disciplines, all involved in caring for elderly cancer patients. In addition to inpatient and outpatient oncologists, we interviewed specialists (gynecologists, surgeons, internists, gastroenterologists, and radiologists) and general practitioners. Thirty-two interviews were held via telephone, and six interviews were held in person at the interviewee’s premises. The interviews ranged from 14 to 55 min, with a median duration of 35 min. Participants’ characteristics are shown in Table 1.

**Table 1:** Characteristics of interviewed physicians

	Oncologists (*N* = 15)	Other specialists (*N* = 13)	General practitioners (*N* = 10)	Total (*N* = 38)
Age (median, [range])	45 [31–57]	48 [31–66]	51 [31–66]	48 [31–66]
*Gender*				
Male	10 (66.7%)	6 (46.2%)	5 (50%)	21 (55.3%)
Female	5 (33.3%)	7 (53.8%)	5 (50%)	17 (44.7%)
*Type of care provided*				
Inpatient	9 (60.0%)	7 (53.8%)	–	16 (42.1%)
Outpatient	6 (40.0%)	6 (46.2%)	10 (100%)	22 (57.9%)

The interviewed physicians agreed that the care of older cancer patients is associated with specific challenges for patients and physicians alike. In their accounts, interviewees identified treatment decisions influenced by comorbidities, the increased need for consultation due to cognitive impairments, and the organization of therapy and follow-up in difficult life circumstances (e.g., widowed and living alone) as being particularly taxing. Most interviewees consider FCGs to be valuable partners in addressing and mitigating these challenges. The perspectives on the roles of FCGs in the therapy process, however, were heterogeneous, and we identified five general themes (see Table 2).

**Table 2:** Overview of themes regarding FCG involvement in physicians’ perspectives and exemplary implications

1. FCGs are interpreters and translators of medical information
• Physicians’ demands regarding effective communication prompt FCG involvement • FCGs are involved only in selected contexts and their involvement is limited in scope
2. FCGs are providers of organizational and mental support
• FCGs are assigned responsibilities in the course of therapy • FCG involvement is separate from the decision-making situation
3. FCGs give information on the patient and their social context that is potentially relevant to the treatment
• FCGs’ accounts are considered key to sound therapy decision-making • FCGs help physicians gain a realistic picture of patients’ life circumstances (e. g.when patients overestimate their condition and abilities)
4. FCGs are stakeholders with relevant points of view regarding the treatment decision
• FCGs are routinely involved in consultations and therapy decision-making situations • Demand for FCG involvement stems from the notion that FCGs’ capacities and skills help determine treatment outcomes
5. FCGs are individuals who have a disruptive influence on the patient-doctor communication
• FCGs interfere with physician–patient consultations • FCGs must be excluded from decision-making situations

Descriptions of the themes and indicative quotations are presented as follows:

1. FCGs are interpreters and translators of medical information

The notion that one of the essential roles of FCGs is to translate medical information into a form that patients can comprehend was widespread among the physicians interviewed. However, participants broadly acknowledged that it is challenging for patients to absorb highly complex information and process it appropriately, especially in a threatening, high-stress situation where organizational and logistical burdens already have a strong influence on the patient’s circumstances.

*Um, if they [the patients] are well cared for, also by family members, which we often experience here, it is, of course often a logistical achievement for the children to bring them here. Then to constantly do the blood tests, the conversations, so many are then simply overwhelmed with the information and then always have their children with them merely to be able to understand the whole thing. This is often a great challenge for the families.*

*(DB227, inpatient oncologist)*

2. FCGs are providers of organizational and mental support

Physicians interviewed were concerned with creating support networks for their patients. The aim was to organize the patient’s everyday life towards optimal conditions for successful therapy. Furthermore, specific responsibilities were delegated, and relatives were prepared for expected difficulties to be able to react appropriately to these in the course of the treatment. Physicians considered FCGs to be at the center of the patient support network and ascribed them to an intermediary role. An inpatient internist and palliative care provider stated that he always sought to make FCGs part of the “treatment team.” In his experience, patients without committed FCGs are often only poorly cared for.

*The patients we accompany are usually so seriously ill that they can't accompany themselves, can't take care of themselves alone. Um, that means there always has to be someone on site when it's outpatient, um, and therefore those who actively accompany are also actively, uh, included in the team. Um, they also get psychological support if they want it, they are instructed in the care of their patients.*

*(DB 209, inpatient palliative care provider)*

One participant, a general practitioner, emphasized the distinction between “academic medicine”—which he only comes into contact with “through doctors’ letters”—and his kind of medicine, which was more concerned with questions like.

*how do I get him to the toilet? How do I get him to eat? Does he drink enough? And these are typical little things that have to be discussed.*

*(DB110, general practitioner)*

Discussing these aspects with patients and FCGs in the run-up to a therapy decision was also crucial since younger and less experienced physicians may not be as aware of these challenges*:*

*When I talk to people who are at the beginning of their career and who do not think that far, then it is difficult to knock on their door mentally. And then it is, of course, better to have a caregiver with you who can see the problems.*

*(DB110, general practitioner)*

3. FCGs give information on the patient and their social context that is potentially relevant to the treatment

Considering that complex and intensive therapies depend not only on clinical parameters but also on patients’ wishes and needs, physicians queried FCGs about the patients’ life circumstances. For example, doctors only got to see the patient briefly in conversation. Through FCGs, doctors learned that patients “pulled themselves together” to appear healthier and more functional than they were. Based on that impression, physicians might recommend or continue inappropriate therapy for the patient.

*If it’s a close relative, they, of course, know them better and can better assess their living situation. They come to us in the outpatient clinic all dressed up and look very fit. And then they might have gathered all the energy of the whole week to come in. Then I think: ‘He’s totally fit’. […] Then I can’t assess the situation that way. I can certainly do that differently with someone who knows - whether it’s a relative or a neighbour.*

*(DB126, outpatient oncologist)*

The FCG may also express views of the patient that the latter may not have articulated with sufficient clarity. FCGs’ accounts helped determine if specific treatment options could be reasonably implemented or if the chances of success were low due to organizational barriers and other circumstances primarily unknown to the physician.

In some cases, the need for organization went beyond the treatment of the current case, e.g., if the person now diagnosed with cancer can no longer meet the care of their spouse. Addressing these organizational demands goes beyond actual medical tasks. However, this was deemed necessary for implementation and success of the recommended therapy.

*So now I had a case where, um, the woman had always been cared for by her husband and now the husband has a very strong suspicion of pancreatic carcinoma, is in hospital himself and now, um, the, the care of the woman has to be clarified first, even though she is not the oncological patient. [...] Well, and then of course it is important that the, if there are, if the children can then help decide where the patients go, how the care continues, that is not our medical task at all, although one has to think about it.*

*(DB111, inpatient oncologist)*

4. FCGs are stakeholders with relevant points of view regarding the treatment decision

If the views and opinions of trusted persons were seen as potentially useful from the outset, a triad of patient, physician, and FCG was likely to emerge. However, one inpatient oncologist (DB223) instead established multiple dyads (patient-doctor, FCG-doctor, doctor-doctor). This approach is suitable for identifying the FCG’s influence on the patient, although it remained unclear how conflicting views and opinions could be resolved in this constellation.

Another inpatient oncologist introduced the term “family council” when asked about the therapy decision-making process. While he emphasized that patient autonomy must be upheld, therapy decision-making is best discussed with FCGs.

*We offer, in principle: ‘you could ultimately be treated with this therapy in this situation.’ Yes? Whether it is a curative approach or a palliative approach. Um, in the end it is important to me that the patient, if possible, also discusses it in the family council. For me that is, um, the way.*

*(DB109, inpatient oncologist)*

Some participants recognized that cancer therapy decisions could also have far-reaching consequences for FCGs. Therefore, involving them in the therapy decision-making was deemed advisable.

*Um, well, you have to essentially include them [FCGs] in the decision on the potential need for care, because, um, it, uh, often changes the entire family environment, for the patients and for the relatives. This has to be addressed. If this is not done, which also happens sometimes, then, uh, it is more often, uh, a surprised situation, what do I do now, how should it go on ...*

*(DK238, inpatient oncologist)*

5. FCGs are individuals who have a disruptive influence on the patient-doctor communication

While most participating physicians argued that the presence or involvement of an FCG is beneficial to the patient and enables a suitable and appropriate decision for the patient, it was also noted that an FCG’s presence could place pressure on the patient and unduly influence their decision. Patients then might make therapy decisions primarily due to social desirability and as a favor to their FCGs.

*They [FCGs] play a big role because they basically always convey to the patient what he should do. One gets the impression that many patients also like to do therapies for the sake of their caregivers. This is then not quite so, um, verbalised. But sometimes you can sense that. […] And the patient himself, at his advanced age, may sometimes find it too strenuous. But he does go along with it because he doesn’t want to disappoint his relatives.*

*(DB112, outpatient oncologist)*

This view was further intensified and pointed out by one participant who described consultations with FCGs present as particularly stressful. He suspected that FCGs do not always represent the interest of patients.

*sometimes [patients are] actually surrounded by relatives, to have two or three relatives with them. I do include them in the discussion, but I always explicitly address the patient himself and would also discuss the possible therapy decisions with the patient himself and not give anything to whether the caregivers say: we don’t want that, but we do want that. Of course, they sometimes interfere and say: we would like it this way and that way and so and so. But in the end, I always wait for the patient’s decision.*

*(DB224, outpatient oncologist)*

He further raised concern that FCGs might influence the therapy decision for selfish or even harmful motives.

*I would even go so far as to say: “Stop here!” when they say: “Oh, don’t put so much strain on Granny!”. Then I sometimes even say: “So stop, stop here. Granny still decides alone and you don’t.” Because you always have to be able to reproach them a little bit, that maybe they even want grandma to die soon, because maybe she has a nice inheritance or because they are annoyed by grandma. Of course, you don’t want to reproach most of them, for God’s sake.*

*(DB224, outpatient oncologist)*

## Discussion

Following previous findings that FCGs can play an essential role in deciding on therapy, 38 semi-structured interviews with doctors involved in caring for elderly cancer patients were analyzed. We identified five distinct perspectives on FCGs’ roles: (1) FCGs are interpreters and translators of medical information; (2) FCGs are providers of organizational and mental support; (3) FCGs give information on the patient and their social context that is potentially relevant to the treatment; (4) FCGs are stakeholders with relevant points of view regarding the treatment decision; (5) FCGs are individuals who have a disruptive influence on patient-doctor communication.

Although physicians widely recognize the importance of FCGs in the therapeutic process, they predominantly perceive them in this context as passive facilitators and supporters. Recent findings from research on triads of physicians, cancer patients, and FCGs indicate that physicians underestimate the influence of FCG on treatment decisions [33]. Hence, by addressing FCGs in this way, physicians run the risk of not gaining a complete understanding of the treatment decision and its circumstances [34].

While unique in our sample, it is noteworthy that physicians may suspect FCGs of harboring selfish or harmful intentions towards the patient. Irrespective of whether such reservations are justified, it is precisely the case that if an undue influence on the patient and a violation of their autonomy is suspected, this can be recognized and countered through the close involvement of FCG. The skeptical remarks of participant DB224 point towards a general problem of triangulating medical decision-making settings. The strict exclusion of FCGs from any substantial participation in decision-making, on the one hand, elevates the autonomy of the patient to the most important and decisive factor in decision-making, and on the other hand, it ignores the broader context of the patient, which generally includes FCGs. The former is undoubtedly desirable at face value; however, the patient’s life is embedded in the life practice of others and the involvement of family and community in important decision-making may be a central value for the individual. Even from a strictly medical perspective, it can be argued that whether or not a therapy option has the desired outcome can depend on the life circumstances, abilities, and capacities of the FCGs.

Joint efforts to cope with disease can be affected by unresolved communication and relationship issues within the family. Physicians reported that elderly patients often make treatment decisions according to the preferences of their FCGs rather than their own. In this way, they can fulfill expectations that have been placed on them. Especially in these situations, the presence of the FCGs at consultations is vital so that the clinician can determine whether the patient’s or the FCG’s will is being expressed. If the latter is the case, then it is the physician’s responsibility to strengthen the patient’s autonomy against any outer influences, even if they are unintentional.

Efforts have recently been made to develop and evaluate guidelines for handling and integrating FCGs in oncology discussions and decision-making situations [35, 36]. Many German oncology guidelines already recognize the importance of FCGs in cancer-related consultations and recommend their involvement, albeit to different extents [19–23]. However, recommendations to enable strong FCG involvement are followed less often. Physicians who do not follow them risk working with an incomplete picture of the circumstances surrounding the patient’s living conditions, circumstances, and treatment decision. Concerning the context of the patient’s life, the controversial question arises: What is the benefit to the patient of an ostensibly free and autonomous decision if the external factors necessary for realizing the desired outcome (including capabilities and capacities of FCG) are not adequately assessed? The chosen therapy option then may consequently fail given the real-world circumstances.

Recent findings from other patient groups, i.e., individuals with severe mental-health-related illnesses [17], support the notion that FCGs’ close involvement in shared decision-making processes can be beneficial. However, their involvement also has consequences for FCGs. For example, a systematic review has shown that caring for cancer patients is associated, among other adverse effects, with significant reductions in quality of life and psychological distress, which are linked to the development of depression [37]. In addition, FCGs of cancer patients have also reported an increase in unmet needs. These relate to information about care and care planning, support in coping with anxiety when the patient’s physical or mental condition deteriorates, and preparing for the possibility of the patient’s death, as well as one’s own grief. FCGs are thus put at risk of strain and dysfunction [38–41]. Such aspects must be discussed before attempting to involve the FCG more in treatment and consultations.

### Strengths and limitations

By interviewing physicians from various backgrounds and specialities, we gained a broader understanding of perspectives on the involvement of FCGs in the cancer treatment of elderly patients. Due to this variety, the physicians gave their accounts based on different situations in different stages of therapy, including follow-up.

However, studies with a more explicit focus on oncologists and therapy decision-making situations are needed to draw further conclusions about the roles of FCGs in specific decision-making situations. In addition, while our study’s sample is sufficiently large and diverse to represent a variety of perspectives, it still does not allow for stratification to reach robust conclusions about the extent to which characteristics of physicians are related to perspectives on FCG involvement. Thematically focused interview studies are desirable in this regard.

As with all interview studies, a bias concerning the social desirability of the participants’ answers cannot be ruled out, especially considering the topic’s sensitive nature. A study design that preserves the anonymity of the participants towards the researchers could address such reservations.

The study aimed to explore a spectrum of perspectives and gain insight into their possible implications. Quantitative surveys developed from these findings would be suitable to address questions on the frequency of certain attitudes, statements, and actions among doctors.

## Conclusion

The results of this study provide insight into a wide range of physicians’ perspectives on FCG involvement in cancer therapy, consultation settings, and therapy decision-making processes. This provokes debates about cancer therapy and therapy decision-making in old age, such as triadic communication, protection of patient autonomy, and family-centered treatment. These exploratory findings need to be supplemented by further research to anchor corresponding topics in general medical training and continuing professional training.

## Data Availability

The participants’ consent does not cover the distribution of the interview material or the transcripts.

## Appendix

Table

**Table 3:** Mentions of the role of family caregivers in cancer treatment according to major German oncology guidelines

German S3-guideline “Colorectal cancer”	
Mention of FCGs	“Participatory decision making: All recommendations in the guideline should be seen as recommendations, which are made using a participatory decision-making process between physicians and patients and their family.” (p. 28)
Comment/ synopsis	The patient's family is presented in the introduction to the guideline as a decision maker regarding treatment decisions. Furthermore, there is no specific mention of FCGs
German S3-guideline “Breast cancer”*	
Selected mentions of FCGs	“The recommendations of the interdisciplinary guideline are directed at all physicians […] and all women suffering from breast cancer as well as their caregivers.” (p. 29) “The patient's wish to have the conversation or further conversations together with a trusted person (partner, caregivers, patient representatives) should be asked for” (p. 36) “All patients and their caregivers should be informed about the possibilities of psycho-oncological support at an early stage and during the illness. […] caregivers are to be included in the psycho-oncological care” (pp. 251) “Physicians who provide follow-up care should support the involvement of (spouses) partners and caregivers in follow-up” (p. 384)
Comment/ synopsis	Caregivers are given comprehensive consideration in the breast cancer guideline. Doctors are explicitly encouraged to ask for the patient's wishes regarding the involvement of caregivers
German S3-guideline “Lung cancer”	
Selected mentions of FCGs	“The recommendations of the guideline are directed to all physicians […] and to all patients suffering from lung cancer and their caregivers.” (p. 26) “Special attention and awareness of the importance of these talking points is needed to offer the patient and their caregivers an open conversation about […] dying in all dimensions early and repeatedly in the course of the illness. Conversations about dying/death wishes should also be conducted proactively.” (pp. 144) “General measures to alleviate respiratory distress include […] the involvement of caregivers, especially with the aim of reducing hecticness and panic in cases of severe respiratory distress and having a calming effect on the patient” (p. 335) “The patient and his caregivers should be adequately informed about the impending death, the expected changes in the dying phase and the individuality of the process.” (p. 338)
Comment/ synopsis	Caregivers are addressed predominantly in the context of patient symptom management and towards preparedness for the patient’s death
German S3-guideline “Prostate cancer”	
Selected mentions of FCGs	“The options for palliative therapy should be discussed comprehensively and at an early stage with the patient and their caregivers.” (p. 223) "The patient and the caregivers should be able to shape the time of palliative therapy according to their preferences and be comprehensively advised and informed about this according to their wishes" (p. 225) “In order to deal with […] limitations due to the advanced stage of the disease, discussions should be held with the patients and their caregivers, and the possibilities and limits of therapeutic interventions shall be addressed, and should also focus on the appropriate organization of everyday life.” (p. 227)
Comment/ synopsis	Caregivers are mentioned exclusively in the context of late-stage cancer, subsequent palliative care and the patient’s end of life
German S3-guideline “Palliative care”*	
Mentions of FCGs	“In palliative care, the quality of life of the patient affected by incurable cancer and their family carers is of central importance.” (p. 30, English short version) “The following principles shall be applied in palliative care of family carers of a patient with incurable cancer: 1. Respect the needs and distress of the family carers and respond to them; 2. Determine realistic goals of care; 3. Be informed about specific support offers for family carers.” (p. 31, English short version) “Palliative care aims to improve or maintain the quality of life of patients with a life-threatening illness and their caregivers.” (p. 11) “Caregivers and other trusted persons of the patient with a non-curable cancer should be involved in the decision-making process to the extent desired by the patient.” (p. 124)
Comment/ synopsis	In this guideline, FCGs are mentioned throughout the document and are considered with regard to a variety of aspects

^*^These guidelines are not translated into English. The passages cited are translated by the authors

3
